# The effect of physical activity in an alpine environment on quality of life is mediated by resilience in patients with psychosomatic disorders and healthy controls

**DOI:** 10.1007/s00406-018-0930-2

**Published:** 2018-07-25

**Authors:** Cornelia Ower, Georg Kemmler, Theresa Vill, Caroline Martini, Andrea Schmitt, Barbara Sperner-Unterweger, Katharina Hüfner

**Affiliations:** 1 Department of Psychiatry, Psychotherapy and Psychosomatics, University Hospital for Psychiatry I, Medical University of Innsbruck, Anichstr. 35, 6020 Innsbruck, Austria; 2 Department of Psychiatry, Psychotherapy and Psychosomatics, University Hospital for Psychiatry II, Medical University of Innsbruck, Anichstr. 35, 6020 Innsbruck, Austria; 3 Department of Psychiatry and Psychotherapy, University Hospital, Ludwig Maximilians-University (LMU) Munich, Munich, Germany; 4 Laboratory of Neuroscience (LIM27), Institute of Psychiatry, University of Sao Paulo, São Paulo, Brazil

**Keywords:** Alpine environment, Physical activity, Resilience, Psychosomatic disorders, Quality of life

## Abstract

**Objective:**

Physical activity (PA) in an outdoor environment has been shown to exert positive effects on mental well-being beyond those found for PA indoors. The specific effect of an alpine environment has not been investigated so far. Here we evaluate the association of PA in an alpine environment with resilience and quality of life (QOL) in patients with psychosomatic disorders and controls.

**Methods:**

194 patients with psychosomatic disorders (mostly somatoform disorder and major depressive syndrome) and 326 healthy controls were included in this web-based cross-sectional study. PA was scored using an adapted version of the Global Physical Activity Questionnaire including the environmental aspect (indoor, outdoor, alpine environment). Resilience was assessed using the Resilience Scale-13, QOL using the WHOQOL-BREF. Group comparisons, correlation and mediation analyses were performed.

**Results:**

Patients showed significantly lower levels of resilience (*p* < 0.001) and QOL (*p* < 0.001) compared to controls. PA in an alpine environment was associated with resilience (patients: *r* = 0.35, *p* < 0.001; controls *r* = 0.18, *p* < 0.001). There were no significant associations between PA in other environments (outdoor or indoor) and resilience. PA in all three environments correlated with subcategories of QOL. The effect of PA in an alpine environment on QOL was partly mediated by resilience in patients (68% of total effect mediated, *p* < 0.001) and controls (49% mediated, *p* = 0.006).

**Conclusion:**

There is a positive effect of PA in an alpine environment on mental health beyond that of physical activity itself. Preventive and therapeutic programs should thus include physical activity, but also take additional benefits of natural environments into account.

## Introduction

Physical activity (PA) is known to improve physical and psychological well-being [1]. The World Health Organization (WHO) recommends at least 150 min of moderate-intensity physical activity (PA) throughout the week [2]. Reduced physical activity is associated with chronic disease, whereas regular exercise enhances physical and mental health [3]. While the effects of physical activity have been well established in chronic somatic diseases, studies in psychosomatic conditions, especially somatoform disorders, are much less prevalent [4]. PA is known to improve symptoms of depression, anxiety and panic disorder [5] as well as enhance measures of mental well-being like quality of life (QOL) [6] and resilience [7]. Resilience can be viewed as one’s ability to bend but not break, bounce back, and perhaps even grow in the face of adverse life experiences. Determinants of resilience include a host of biological, psychological, social and cultural factors that interact with one another to determine how one responds to stressful experiences [8]. Additionally, people with mental health problems recover more rapidly when regular physical activity is performed [9]. PA, resilience and QOL have been shown to be interconnected: There is a direct effect of PA on QOL and an indirect effect mediated by resilience [7, 10].

Benefits of PA are even greater when performed outdoors: For healthy individuals, there is evidence that exercising outdoors results in greater improvements of mental well-being than exercising indoors with greater feelings of delight, energy and revitalization, as well as decreases in frustration, tiredness and anger [11]. Additionally, in healthy subjects, contact with nature is known to improve overall life-satisfaction [12], the feeling that one’s life is worthwhile [13], cognitive functioning [14] and QOL [15]. Physical activity in outdoor, natural environments is linked with improvements in social networking and feelings of connectivity and companionship, an increased appreciation of nature, improvements in self-esteem and a means of escape from modern life [11]. The protective effect of physical activity against depression and suicidal ideation has been shown to be mediated by self-esteem and social support not the activity per se [16]. Additionally, outdoor physical activity shows higher adherence rates [17], and the outdoor environment seems to promote physical activity [18]. By building a personal bond to individual mountain sides the positive impact of the outdoor environment on mental well-being is enhanced [19]. Due to urbanization, a lack of contact with nature affects city dwellers which is associated with a higher prevalence for psychiatric disorders like mood and anxiety disorders [20].

A limiting factor in the available studies on the effect of outdoor, natural environments is that mostly subjects without overt mental health disorders were studied, and the outdoor environments mostly included parks or forests [21]. Only few studies have examined the effect of outdoor physical activity in patients with mental health disorders. It is known that patients with mild to moderate depression feel more active after PA outdoor than indoor [22]. Exercise in green spaces (e.g., forest, countryside) improves both self-esteem and mood irrespective of duration, intensity or mental health status [23].

The effect of PA in an outdoor alpine environment on mental health has rarely been researched to date. In a pilot study the therapeutic benefit of the alpine environment was assessed using a mountain hiking paradigm in a sample of 17 suicidal patients. Results indicate a significant reduction in depression, hopelessness and suicidal ideation as well as an improvement in QOL [24]. A comparison with other environments (outdoor, indoor) was not performed. Nevertheless, the results suggest therapeutical benefits of PA in an alpine environment in the treatment of patients with mental health disorders.

To the best of our knowledge, there is no study assessing the benefit of PA in an alpine environment in comparison with the effects of PA in outdoor and indoor environments on mental health. The aim of the current study was to find out if there are beneficial effects of outdoor PA which go beyond that of PA itself, and if the effects might be even greater in an alpine setting compared to urban outdoor environments such as parks. In the present study we investigate the effect of PA in an alpine environment, in an outdoor (non-alpine) environment and indoors and their association with resilience and QOL in patients with psychosomatic disorders and controls. We also assess a possible interaction between the three components PA, resilience and QOL using a mediation analysis.

## Methods

### Study design

**Fig. 1:**
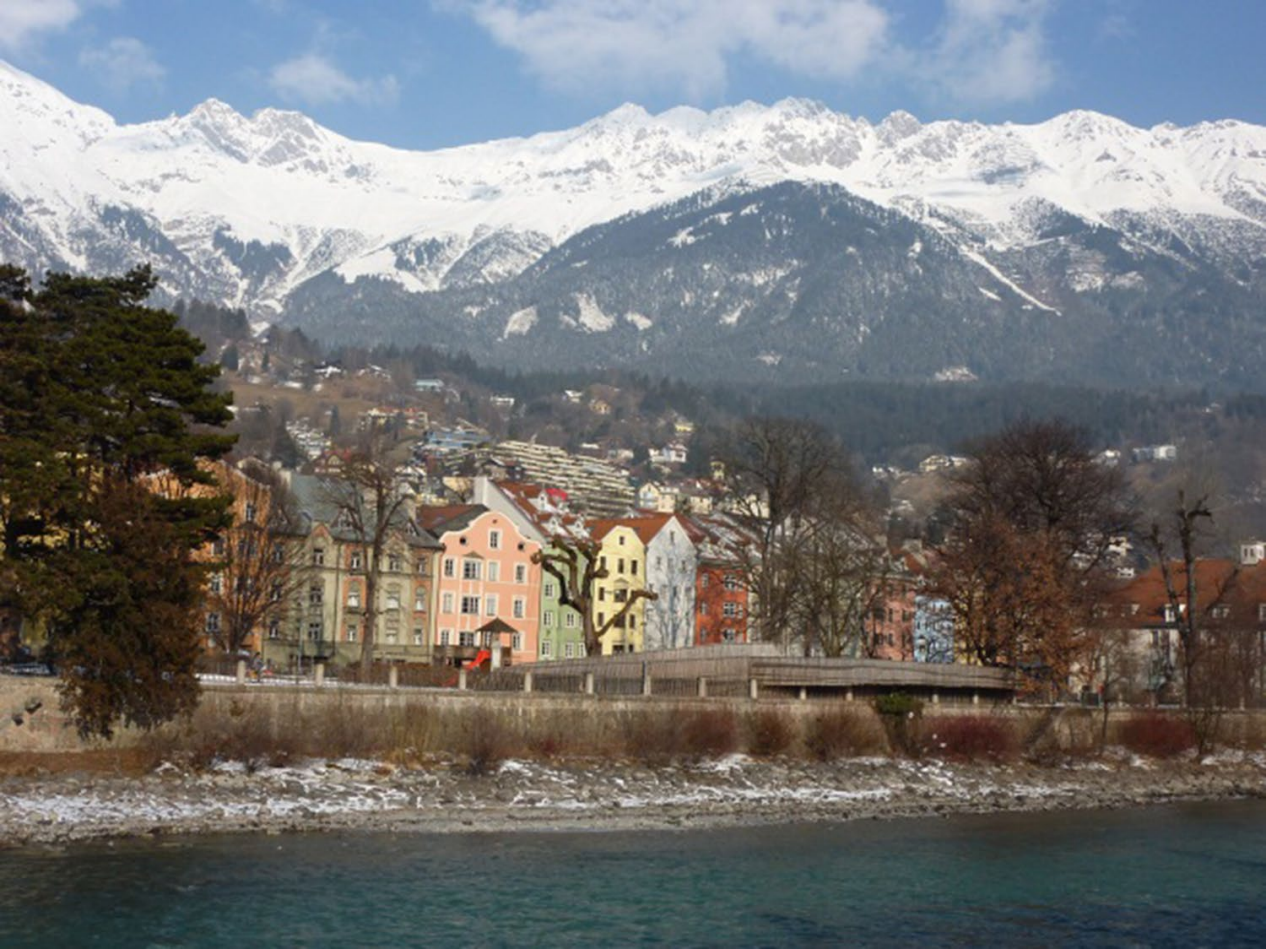
Photo of the city of Innsbruck showing the close proximity between the urban space and the high mountain alpine environment. The alpine environment can be accessed on foot, bike or skis as well as using a car, bus or cable car directly from the city center

This is a cross-sectional observational study. The current data are part of a larger study examining the effect of PA in an alpine environment on mental health. Innsbruck is one of the few urban spaces located directly within the Alps and thus allows for easy access to the alpine environment (Fig. 1). The ethics commission of the Medical University of Innsbruck reviewed and approved the study protocol. After being informed in detail about the study aims and procedures, participants provided informed consent prior to study participation. Study recruitment was conducted over a 4-month period in 2016.

### Participants

**Fig. 2:**
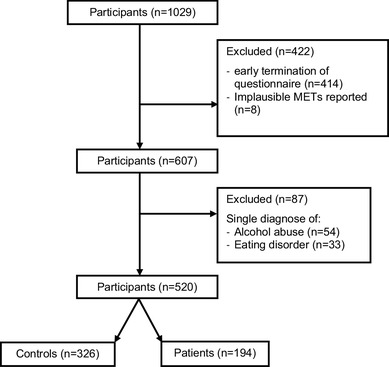
Flowchart of patient and healthy control recruitment *Early termination of the questionnaire was defined as termination of the questionnaire prior to completion of the items sociodemographics, Patient Health Questionnaire, Physical activity and Resilience

A total of 1029 participants were invited to an open online survey via email, social media and classified websites or recruited whilst treated at the Innsbruck University hospital at the inpatient or outpatient clinic. For the present analysis participants who terminated the questionnaire early were excluded [this was defined as: individuals who did not complete at least questions on sociodemographics, Patient Health Questionnaire, Physical activity and Resilience (*n* = 414)]. Skipping questions was not possible in this questionnaire. Additionally individuals with implausible PA values (*n* = 8) were excluded from the study. Individuals who screened positively for alcohol abuse only (*n* = 54) or for an eating disorder only (using PA for losing weight: Anorexia nervosa, Bulimia nervosa; *n* = 33) were excluded from further analysis (Fig. 2). The 520 participants consisted of two groups: Patients screened positively for mental health disorder on the Patient Health Questionnaire (PHQ, *n* = 194) which uses DSM-IV criteria. Two participants reported a mental health disorder not screened for in the PHQ and were, therefore, classified as patients. Participants without positive PHQ screening (*n* = 326) built the control group (HC).

### Measures

Sociodemographic parameters included information on age, sex, education and marital status. Mental health was assessed using the German version of Patient Health Questionnaire (PHQ), which screens for somatoform disorders, depressive disorders, anxiety disorders, eating disorders and alcohol abuse. We used the PHQ as categorical non-dimensional screening instrument [25]. Additionally, open text fields were provided for entering psychiatric diagnoses. Resilience was measured using the German “Resilienzskala—RS13” which is a short version of the original RS -25. The minimum score is 13 and maximum score is 91 with values between 65 and 73 representing moderate resilience, values below indicate low resilience and values above high resilience [26]. Quality of life was measured with the WHOQOL-BREF, a 26-question short version of the original WHOQOL-100. Four domains of QOL are assessed: “physical health”, “psychological”, “social relationships” and “environment”. Two questions reflect overall QOL and general health. The four domains are calculated with mean values of the relevant items multiplied by four to ensure comparability with the WHOQOL-100 [27]. PA was assessed using the Global Physical Activity Questionnaire (GPAQ-2) of the WHO which is an international standardized tool [28] to measure self-reported PA in a retrospect of a typical week. PA is calculated using metabolic equivalents (METs) as a unit for energy use. Moderate-intensity activities are assigned a value of 4 METs; vigorous-intensity activities are assigned a value of 8 METs. We used the official scoring protocol [29] to calculate total moderate-to-vigorous PA in MET minutes/week. In addition to the standard questionnaire, we used a modified version to identify PA performed in different environments (indoor, outdoor, and alpine environment). We defined “PA in an alpine environment” as PA which involves overcoming vertical energy in a natural environment which was worded as “PA performed on the mountain” in the questionnaire. For each environment we gave multiple examples of PA that would be characteristic for the respective environment, e.g., alpine: ski touring, skiing, tobogganing, alpine trail running, mountain hiking, mountain biking; outdoor non-alpine: rollerblading, soccer on an outdoor field, swimming in an outdoor pool, horse riding outdoors; indoor: cardio exercises in a fitness studio, indoor basketball, indoor yoga, indoor dancing.

### Statistical methods

Prior to the analysis, metric variables were checked for deviations from normality by assessing their skewness, considering values > 0.5 or < − 0.5 as deviations from a symmetric distribution requiring non-parametric testing. Group comparisons (patients vs. controls) with respect to sociodemographic and clinical variables were performed by means of *t* test, Mann–Whitney *U* test and Chi-square test, depending on the variable type (normally distributed, non-normally distributed metric variables, and categorical variables, respectively). Group comparisons regarding PA, resilience, and quality of life were conducted using *t* test or Mann–Whitney *U* test (again depending on the variable type). The relationship between PA and resilience and QOL was investigated on a descriptive level by means of correlation analysis. Spearman rank correlation coefficients were used as most the variables involved showed deviations from a normal distribution.

To investigate the relationship between PA, resilience and QOL in more detail several mediation analyses were performed based on the approach proposed by Preacher and Hayes using the SPSS macro PROCESS [30]. The model chosen was motivated by findings of Ho et al. who reported that resilience mediated the relationship between PA and mental well-being, assessed with the respective component score of the SF-12, in a sample of adolescents [7]. Building on this, we considered PA as the independent variable, QOL (WHOQOL total score) as the dependent variable, and resilience (RS-13) as a potential mediator between the two variables. Prior to the analysis, the variable PA was log-transformed to obtain a more symmetrical distribution. Separate mediation models for patients and controls were fitted. Both total PA and outdoor PA in alpine surroundings were considered. Effect sizes of total, direct and indirect effects were reported as standardized regression coefficients. Moreover, the relative size of the mediation effect was expressed as a fraction of the total effect (percentage mediated). Following Kenny we used the terms partial and complete mediation if the percentage mediated fell below 80% or lay above this cut-off value [31].

## Results

### Sociodemographic characteristics and clinical features

The sociodemographic characteristics of the total sample are shown in Table 1. The gender distribution was similar between the groups (Chi-square test, *p* = 0.273) as well as city size of residence (Chi-square test, *p* = 0.274). Patients had a higher mean age (Mann–Whitney *U* test, *p* = 0.02), lower level of education (Chi-square test, *p* < 0.001), less frequently employed (Chi-square test, *p* < 0.001), had a lower income (Chi-square test, *p* < 0.001) and were more often single (Chi-square test, *p* = 0.003) than HC.

**Table 1:** Sociodemographic characteristics of patients and healthy controls

Variable	Groups		Comparison		
	Patients (*n* = 194)	Controls (*n* = 326)	Test statistics	*Df*	*p* value
Age in years^a,b^	35.9 ± 12.8	32.9 ± 11.8	*Z* = 2.30		0.021
Female gender^c^	126 (64.9)	196 (60.1)	*χ*^2^ = 1.20	1	0.273
Regular psychiatric medication^c^	68 (35.1)	8 (2.5)	*χ*^2^ = 73.25	1	< 0.001
Education^c^	–	–	*χ*^2^ = 57.51	3	< 0.001
University	46 (23.7)	115 (35.3)		–	–
Secondary school	67 (34.5)	138 (42.3)		–	–
Vocational training	54 (27.8)	35 (10.7)		–	–
Compulsory school and other	27 (13.9)	38 (11.7)		–	–
Marital status^c^	–	–	*χ*^2^ = 11.98	2	0.003
Single	113 (58.2)	199 (61)		–	–
Married	58 (29.9)	114 (35)		–	–
Separated/divorced/widowed	23 (11.9)	13 (4)		–	–
Employment^c^	–	–	*χ*^2^ = 67.91	2	< 0.001
Full-/part-time employment	80 (41.2)	184 (56.4)			
In education/study/vocational training	53 (27.3)	126 (38.7)			
Unemployed	61 (31.4)	16 (4.9)			
Income in euros^c^	–	–	*χ*^2^ = 19.85	3	< 0.001
< 1000	83 (42.8)	99 (30.4)			
1000–2000	60 (30.9)	81 (24.8)			
2000–4000	44 (22.7)	106 (32.5)			
> 4000	7 (3.6)	40 (12.3)			
City size of residence in n of inhab^c,d^	–	–	*χ*^2^ = 2.987	2	0.225
< 10,000	62 (32.0)	105 (32.2)			
10,000–130,000 (eg. Innsbruck)	109 (56.2)	165 (50.6)			
> 130,000	23 (11.9)	56 (17.2)			

^a^Mean ± standard deviation

^b^16.7% missing values

^c^Absolute number (percent)

^d^Number of inhabitants

The most common diagnoses in the patient group were somatoform disorders (54.6%) and major depressive syndrome (38.7%) (Table 2). One hundred (51.6%) patients were diagnosed with more than one mental health disorder, the most common combinations were somatoform disorder and major depressive syndrome (*n* = 45/23.2%).

**Table 2:** Psychiatric diagnoses according to PHQ screening

Mental health disorder of patients (*n* = 194)	*N*/(%)^a^
Somatoform disorder	106/(54.6)
Major depressive syndrome	75/(38.7)
Other depressive syndrome	35/(18.0)
Panic syndrome	36/(18.6)
Other anxiety syndrome	46/(23.7)
Alcohol abuse	32/(16.5)
Binge eating disorder	24/(12.4)
Bulimia nervosa	10/(5.2)
Others	2 (0.4)

^a^Sums exceed 100% because of multiple diagnosis

### Comparison of PA in different environments between patients and HC

Patients and HC did not differ significantly on PA in closed rooms or buildings (PA indoor; Mann–Whitney *U* test, *p* = 0.060; Table 3). The control group had significantly higher values than the patient group regarding overall PA (Mann–Whitney *U* test, *p* = 0.009), PA in an alpine environment (PA alpine; Mann–Whitney *U* test, *p* < 0.001) and PA in an outdoor, non-alpine environment (PA outdoor; Mann Whitney *U* test, *p* < 0.001).

**Table 3:** Comparison of scores of resilience, quality of life and physical activity between patients and HC

Variable	Groups		Comparison	
	Patients (*N* = 194) Mean ± SD	Controls (*N* = 326) Mean ± SD	Test statistics	*p* value^b^
RS -13 score	57.3 ± 16.8	72.8 ± 9.0	*t* = − 13.70	< 0.001
WHOQOL-BREF^a^ score in points				
Total	13.4 ± 2.9	16.9 ± 1.6	*Z* = − 13.27	< 0.001
Physical	13.5 ± 3.6	17.8 ± 1.6	*Z* = − 14.01	< 0.001
Psychological	12.0 ± 3.7	16.6 ± 2.0	*Z* = − 13.21	< 0.001
Social	12.6 ± 4.2	15.7 ± 2.9	*Z* = − 8.24	< 0.001
Environmental	15.4 ± 2.7	17.4 ± 1.7	*Z* = − 8.33	< 0.001
PA in MET min/week				
Overall	5931.0 ± 6805.6	6702.1 ± 6449.0	*Z* = − 2.63	0.009
Alpine	1927.8 ± 3212.6	2733.4 ± 3120.7	*Z* = − 4.74	< 0.001
Outdoor	1007.2 ± 1578.2	1285.0 ± 1739.4	*Z* = − 3.22	< 0.001
Indoor	2996.0 ± 5170.0	2683.7 ± 3927.7	*Z* = + 1.88	0.060

*RS -13* resilience scale 13, *WHOQOL-BREF* WHO quality of life-short version, *PA* physical activity, *MET* metabolic units, *min* minutes

^a^3.7% missing data

^b^*p* values were calculated with Chi-square test for categorical variables and Mann–Whitney *U* test for continuous variables

To assure congruence of the results in the patient population across different diagnostic groups we performed comparisons between the most prevalent mental health disorders diagnosed in the study (somatoform disorders, major depressive syndrome and other anxiety syndromes, Table 2). Patients who screened positively for either somatoform disorder or major depressive syndrome did not differ significantly on total PA (Mann–Whitney *U* test, *p* = 0.143) nor on PA in the three investigated environments (Mann–Whitney *U* test, all *p*s > 0.05) Similar results were found for the comparison of patients with somatoform disorder and those with other anxiety syndromes (Mann–Whitney *U* test, all *p*s > 0.05).

### Comparison of resilience and QOL in patients and HC

The mean score of the RS-13 was significantly lower in patients than in HC (*t* test, *p* < 0.001; Table 3). Additionally, low resilience—represented by a score of less than 65—was measured more often in patients than HC (Chi-square test, *p* < 0.001). Likewise, the total score and all subscores of the WHOQOL-BREF were significantly lower in patients than in HC (Mann–Whitney *U* test, *p* < 0.001).

### Correlation between PA, resilience and QOL

Resilience correlated significantly with PA alpine (patients: *r* = 0.35, *p* < 0.001 and HC *r* = 0.18, *p* = 0.001), but not with PA outdoor or PA indoor in patients and HC (Table 4). In patients QOL total score and all subscales correlated with PA alpine and the same held true for HC, except that in HC the QOL environmental domain did not correlate with PA alpine (Table 4). In patients, not so in HC, several additional correlations between QOL subscores and PA outdoor and PA indoor were found (Table 4).

**Table 4:** Correlations between physical activity, resilience and quality of life in patients with psychosomatic disorders and controls

	PA alpine	PA outdoor	PA indoor
**Controls (** ***n*** ** = 326)**			
RS-13 score			
*r*	0.18**	0.08	0.09
*p*^a^	0.001	0.15	0.10
WHOQOL-BREF score^b^			
Total			
*r*	0.16**	0.08	0.04
*p*	0.01	0.14	0.48
Physical health			
*r*	0.11**	0.06	0.03
*p*	0.05	0.30	0.65
Psychological			
*r*	0.13**	0.07	0.02
*p*	0.02	0.22	0.78
Social relationships			
*r*	0.12**	0.07	0.04
*p*	0.03	0.23	0.52
Environmental			
*r*	0.07	0.03	0.03
*p*	0.23	0.63	0.56
**Patients (** ***n*** **= 194)**			
RS-13 score			
* r*	0.35***	0.11	0.14
* p*	< 0.001	0.12	0.06
WHOQOL-BREF score^b^			
Total			
*r*	0.31***	0.15*	0.16*
*p*	< 0.001	0.04	0.03
Physical health			
*r*	0.36***	0.14	0.18**
*p*	< 0.001	0.05	0.02
Psychological			
*r*	0.28***	0.06	0.15**
*p*	< 0.001	0.38	0.04
Social relationships			
*r*	0.20**	0.19**	0.15**
*p*	0.01	0.01	0.04
Environmental			
*r*	0.23***	0.10	0.09
*p*	< 0.001	0.17	0.22

*RS -13* resilience scale 13, *WHOQOL-BREF* WHO quality of life-short version, *PA* physical activity, *r* correlation coefficient, *p p* value

*p < 0.05, **p < 0.01, ***p < 0.001

^a^*p* values were calculated using Spearman Rank correlations

^b^3.7% missing data

### Mediation analyses

**Fig. 3:**
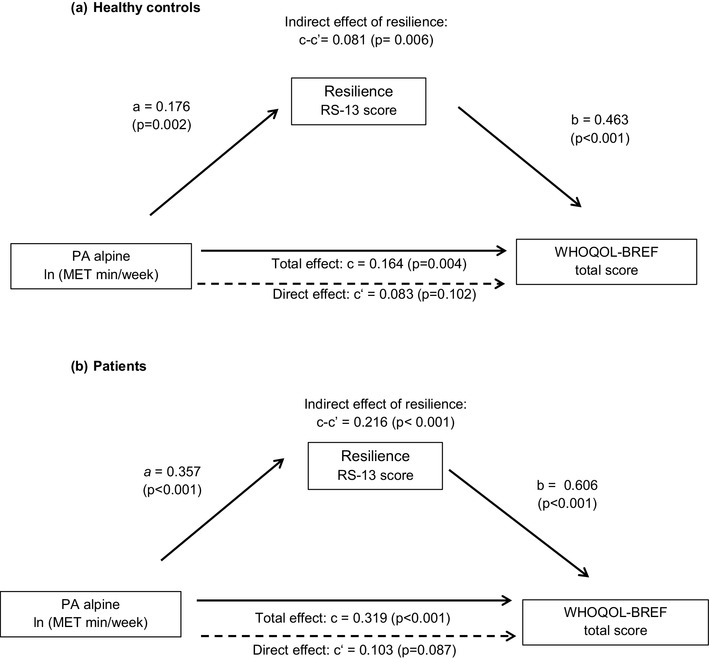
Effect of resilience on the relationship between physical activity in an alpine environment and QOL—results of mediation analysis. Numbers shown in the diagram are standardized regression coefficients. Solid lines indicate statistically significant effects, dashed lines indicate non-significant effects. *MET* metabolic equivalent, *Min* minutes, *PA alpine* physical activity in an alpine environment, *WHOQOL-BREF* World Health Organization Quality of Life Score-short form. *a* = effect of PA alpine on the mediator RS-13 score. *b* = effect of resilience (RS-13 score) on WHOQOL-BREF total score, adjusted PA alpine. *c* = total effect of PA alpine on WHOQOL-BREF total score. *c*′ = direct effect of PA alpine on WHOQOL-BREF total score, after adjusting for resilience (RS-13 score). *c* − c’: Indirect effect of resilience on the relationship between PA alpine and WHOQOL-BREF total score

**Fig. 4:**
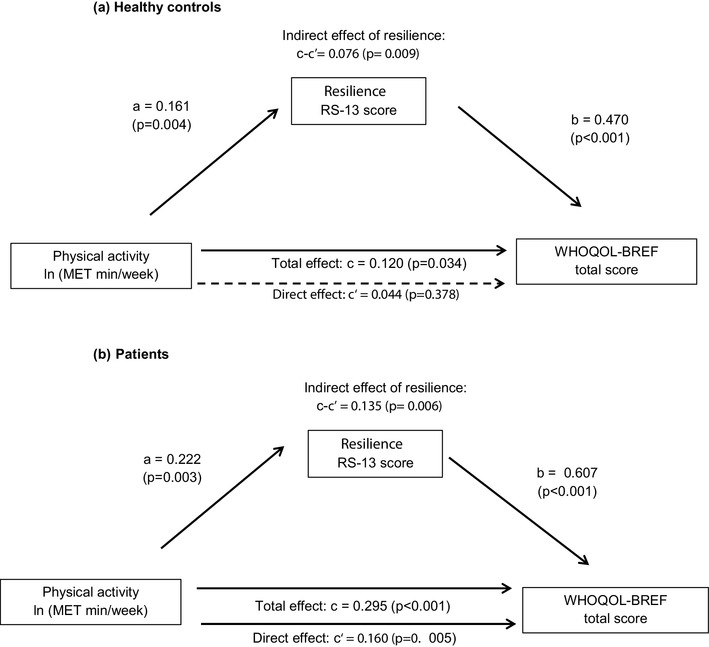
Effect of resilience on the relationship between total physical activity and QOL—results of mediation analysis. Numbers shown in the diagram are standardized regression coefficients. Solid lines indicate statistically significant effects, dashed lines indicate non-significant effects. *WHOQOL-BREF* World Health Organization Quality of Life Score-short form, *MET* metabolic equivalents, *Min* minutes. *a* = effect of physical activity on the mediator RS-13 score, *b* = effect of resilience (RS-13 score) on WHOQOL-BREF total score, adjusted for PA, *c* = total effect of physical activity on WHOQOL-BREF total score, *c*′ = direct effect of physical activity on WHOQOL-BREF total score, after adjusting for resilience (RS-13 score), *c* − *c*′: indirect effect of resilience on the relationship between physical activity and WHOQOL-BREF total score

The relationship between PA, resilience and QOL was investigated in more detail by mediation analysis. Findings are shown in Fig. 3 (PA alpine as the independent variable) and Fig. 4 (total PA as the independent variable). Mediation analysis in HC revealed a significant total effect of PA alpine on participants’ QOL (c = 0.164, *p* = 0.004; Fig. 3a**)**. This effect was partly mediated by resilience (*c* − *c*′ = 0.081, *p* = 0.006) amounting to a proportion of 49% of the total effect. The remaining direct effect of PA alpine on QOL lost its significance (*c*′ = 0.083, *p* = 0.106). However, as the direct effect represented 51% of the total effect there was no indication of complete mediation. Mediation analysis in patients also yielded a highly significant total effect of PA alpine on QOL (*c* = 0.319, *p* < 0.001; Fig. 3b). Again, this effect was partly mediated by resilience. The size of the total effect was reduced by approximately 68% through the mediation (from *c* = 0.319 to *c*′ = 0.103). The remaining direct effect of PA alpine on QOL attained only trend-level significance (*p* = 0.087).

Results for total PA were broadly similar to those for PA alpine (Fig. 4a, b). However, here the proportion of the total effect attributable to mediation by resilience was larger in the control group (63% of the total effect) than in the patient group (46% of the total effect). In the latter group the direct effect (i.e., the part of the total effect not attributable to the mediator) now reached statistical significance (*p* = 0.005).

## Discussion

In the present study we evaluated the association of physical activity in an alpine environment with resilience and quality of life in patients with psychosomatic disorders. The major findings were: (1) Patients with psychosomatic disorders had lower values in resilience and QOL compared to HC, (2) PA alpine, but not in other environments, correlated with resilience in patients and HC, (3) PA in all three environments correlated with QOL in patients and HC and (4) Resilience was found to partially mediate the effect of PA alpine on QOL in patients and HC.

### Resilience and QOL in patients with psychosomatic disorders

We found that patients with psychosomatic disorders display lower levels of resilience and QOL. This confirms previous studies showing that patients with mental disorders have impaired levels of resilience [32] which improve within the process of recovery [33]. Likewise, QOL has been shown to be reduced in patients with mental disorder [34]. This could be due to the fact that both, resilience and QOL are influenced by similar factors such as social support [35], coping strategies in stressful situations [36] and self-respect [10] which can be impaired in patients with psychosomatic disorders. Additionally, certain personality factors have been found to be connected to resilience as well as QOL [37, 38].

### Association of PA with resilience and QOL

The link between PA and QOL is well known though it has rarely been evaluated in a population with psychosomatic disorders. In patients with depression physical and psychological subdomains of QOL show improvements following regular exercise [39], However, most outpatients with mental disorders do not reach recommended levels of PA [40]. Theories on the mechanisms through which physical activity could influence QOL have been generated, and much is based on psychoimmunological aspects. Multiple large studies [41, 42] have consistently shown that there is an inverse, independent, dose–response relation between level of PA and immune cytokines (e.g., IL-6 and CRP), while depression is associated with increased plasma levels of immune cytokines [43].

PA not only influences QOL, but there is also a connection with resilience, which has been demonstrated for healthy subjects [44] as well as individuals with depression [45] or cancer [46]. The relationship between the PA and resilience seems to be non-linear, in contrary, there might be an optimal amount of PA for building resilience [44]. In the present study, we could identify resilience as a mediator between PA and QOL, consistent with findings from previous studies [7, 10].

### The effect of the alpine environment

The effect of PA in an outdoor alpine environment on mental health has rarely been researched to date. In the present study exercise in the alpine environment was the only one associated with resilience, while this association was not found for PA in an outdoor non-alpine environment or for PA indoors. The principal feasibility of a program including physical activity in an alpine environment has been demonstrated in a group of patients with mixed psychiatric disorders (mostly depression, emotionally instable personality disorder, and substance abuse) who participated in a mountain hiking program [24]. Other studies have evaluated the benefit of “outdoor adventure therapy” in patients with mental illnesses. Following activities like mountain biking, raft building and group exercises, adults and youth with mental illness experienced significant improvements in self-esteem, mastery and resilience [47, 48]. However, in these studies the adventure therapy comprised therapeutic sessions in addition to the outdoor activities and the activities took place in mixed environments, a differential analysis of the diverse environments for PA is missing.

The American Psychological Association suggests 10 ways to build resilience, which includes maintaining a good relationship with friends and/or family, advice on stress management, moving towards ones goals, taking decisive actions in adverse situations, looking for opportunities of self-discovery, developing self-confidence and to exercise regularly [49]. Looking at this agenda, physical activity in an alpine environment seems an ideal measure joining those points in one single process. During physical activity in an alpine environment also many factors not related to the exercise per se, and more related to aspects of companionship, self-esteem, pursuing ones’ goals, risk management and contact to nature come into play. This is important since nature experiences (from gardening to wilderness activities) increase a sense of healthy internal locus of control and self-efficacy [50]. Programs which include outdoor activities have been found to be effective through nature itself [51]. A study from England compared different outdoor environments: greater connectedness to nature and restoration was observed following visits to rural and coastal locations compared with urban green space, and to sites of higher environmental quality (operationalized by protected/designated area status, for example, nature reserves) [52]. During PA in an alpine environment moderate manageable risks are encountered which have to be overcome, a process which increases resilience resulting in increased QOL [7]. Additionally PA alpine often requires a high level of social interaction which could provide social support and improve resilience as well as mental health problems [7, 45].

### Limitations

The main limitation of the study is that in a survey study no causal relationship between the physical activity measures and the psychometric assessments can be obtained. However, this is inherent to this kind of study and can only be addressed in a prospective intervention study. To minimize the distorting effect of the reported self-perception, we used questionnaires like the Global Physical Activity Questionnaire which are established worldwide and showed good validity and reliability [28]. We did not record whether individuals were undergoing psychotherapy and there might also be an effect of psychiatric medication on resilience and QOL. The present study does not allow the differentiation which components of the alpine environment lead to the observed positive effects, this should be addressed in a follow-up intervention study.

## Conclusion

Physical activity in an alpine environment correlates with resilience and QOL in patients with psychosomatic disorders and healthy controls. Since alpine environments are regionally limited other natural settings should be explored as an alternative. To identify more specifically the factors comprising the positive effect of the alpine environment a prospective study comparing the effects of an alpine environment with those of other environments should be performed. To better underpin the effectiveness of PA in an alpine environment a dose–response relationship should ideally be demonstrated [53].While these scientific questions remain to be addressed we propose from a practical perspective that the shown positive effects of the alpine environment should be used in everyday clinical practice: Therapies should not only include gym-based programs or classical Nordic walking in streets or yards, but take the additional effects of outdoor natural environments into account.
